# The Pathogenesis of Cardiomyopathy in Friedreich Ataxia

**DOI:** 10.1371/journal.pone.0116396

**Published:** 2015-03-04

**Authors:** Arnulf H. Koeppen, R. Liane Ramirez, Alyssa B. Becker, Sarah T. Bjork, Sonia Levi, Paolo Santambrogio, Patrick J. Parsons, Pamela C. Kruger, Karl X. Yang, Paul J. Feustel, Joseph E. Mazurkiewicz

**Affiliations:** 1 Research, Neurology, and Pathology Services, Veterans Affairs Medical Center, Albany, New York, United States of America; 2 Departments of Neurology and Pathology, Albany Medical College, Albany, New York, United States of America; 3 Research Service, Veterans Affairs Medical Center, Albany, New York, United States of America; 4 San Raffaele Scientific Institute, Milan, Italy; 5 Vita-Salute San Raffaele University, Milan, Italy; 6 Division of Environmental Health Sciences, Wadsworth Center, New York State Department of Health, Albany, New York, United States of America; 7 Department of Environmental Health Sciences, University at Albany, Albany, New York, United States of America; 8 Center for Neuropharmacology and Neuroscience, Albany Medical College, Albany, New York, United States of America; Lady Davis Institute for Medical Research/McGill University, CANADA

## Abstract

Friedreich ataxia (FA) is an autosomal recessive disease with a complex neurological phenotype, but the most common cause of death is heart failure. This study presents a systematic analysis of 15 fixed and 13 frozen archival autopsy tissues of FA hearts and 10 normal controls (8 frozen) by measurement of cardiomyocyte hypertrophy; tissue frataxin assay; X-ray fluorescence (XRF) of iron (Fe) and zinc (Zn) in polyethylene glycol-embedded samples of left and right ventricular walls (LVW, RVW) and ventricular septum (VS); metal quantification in bulk digests by inductively-coupled plasma optical emission spectrometry (ICP-OES); Fe histochemistry; and immunohistochemistry and immunofluorescence of cytosolic and mitochondrial ferritins and of the inflammatory markers CD68 and hepcidin. FA cardiomyocytes were significantly larger than normal and surrounded by fibrotic endomysium. Frataxin in LVW was reduced to less than 15 ng/g wet weight (normal 235.4±75.1 ng/g). All sections displayed characteristic Fe-reactive inclusions in cardiomyocytes, and XRF confirmed significant regional Fe accumulation in LVW and VS. In contrast, ICP-OES analysis of bulk extracts revealed normal total Fe levels in LVW, RVW, and VS. Cardiac Zn remained normal by XRF and assay of bulk digests. Cytosolic and mitochondrial ferritins exhibited extensive co-localization in cardiomyocytes, representing translational and transcriptional responses to Fe, respectively. Fe accumulation progressed from a few small granules to coarse aggregates in phagocytized cardiomyocytes. All cases met the “Dallas criteria” of myocarditis. Inflammatory cells contained CD68 and cytosolic ferritin, and most also expressed the Fe-regulatory hormone hepcidin. Inflammation is an important factor in the pathogenesis of FA cardiomyopathy but may be more evident in advanced stages of the disease. Hepcidin-induced failure of Fe export from macrophages is a likely contributory cause of damage to the heart in FA. Frataxin replacement and anti-inflammatory agents are potential therapies in FA cardiomyopathy.

## Introduction

Friedreich ataxia (FA) is an autosomal recessive disorder that is best known for its disabling neurological phenotype. The most common cause of death, however, is cardiomyopathy [1].

Friedreich [2] described hypertrophy and discoloration of the myocardium in 3 of his initial 6 patients with fatal course but did not consider the heart lesion part of the pathological phenotype. Eighty years later, Russell [3] established that chronic myocarditis in FA is an integral part of the disorder and stressed that the destructive process was focal and progressed in a piecemeal manner. The current report presents systematic observations on archival autopsy specimens that support myocarditis as an important mechanism in the pathogenesis of FA cardiomyopathy. The work confirms severe reduction of cardiac frataxin levels and the importance of iron (Fe), cytosolic and mitochondrial ferritins [4–6], and the iron-regulatory peptide hormone hepcidin.

## Material and Methods

### Clinical data and specimens

The Institutional Review Board of the Veterans Affairs Medical Center, Albany, NY, USA, has approved this work. For all autopsy specimens, the corresponding author (AHK) has obtained formal written informed consent from the deceased patient's next-of-kin. The consenting process covered the collection of personal health information, permission to process specimens for research purposes, and an option to share collected tissues with other investigators of hereditary ataxia. The archival material consisted of fixed and frozen autopsy specimens of 41 patients with FA. Fifteen were suitable for mapping of Fe and zinc (Zn) and *in situ* quantitative X-ray fluorescence (XRF) of left ventricular wall (LVW), right ventricular wall (RVW), and ventricular septum (VS) because they were stored at 4°C in a sodium phosphate-buffered 4 percent formaldehyde solution (pH 7.4) for less than 2 weeks prior to embedding in polyethylene glycol (Table 1). Specimens that were exposed to fixatives for longer periods were excluded because metals are known to diffuse away over time from their natural sites in the tissue [7].

**Table 1:** Basic clinical data of FA patients and normal controls.

Patient	Sex	Age of onset (years)	Age of death (years)	Cause of death (clinical diagnosis)	Heart weight (g)	GAA1	GAA2
FA patients (15)							
**FA1**	M	4	37	Cardiomyopathy	419	674	674
**FA2**	F	5	28	Cachexia	325	800	1100
**FA3**	F	6	23	Endocarditis	358	668	864
**FA4**	F	7	28	Cachexia	362	681	837
**FA5**	M	7	35	Cardiomyopathy	324	750	1000
**FA6**	M	7	34	Cardiomyopathy	418	1114	1114
**FA7**	M	8	27	Cardiomyopathy	413	700	1070
**FA8**	M	9	40	Cardiomyopathy	660	519	647
**FA9**	M	9	33	Cardiomyopathy	421	925	925
**FA10**	M	10	24	Cardiomyopathy	565	700	1050
**FA11**	M	11	15	Heart failure after scoliosis surgery	384	800	1100
**FA12**	F	15	69	Brain embolism	359	568	568
**FA13**	F	17	50	Cardiomyopathy	487	515	1122
**FA14**	F	18	63	Cardiomyopathy	440	639	730
**FA15**	F	20	48	Cardiomyopathy	436	Not available
**Mean±S.D.** ^a^	M, 8; F, 7	10.2±5	34.6±15.5		425±90		718±159	914±195
**Normal controls (10)**							
**Mean±S.D.** ^a^	M, 5; F, 5		58.6±8.3		445±114		

Abbreviation: ^a^S.D., standard deviation

In 13 of the 15 acceptable autopsy cases, tissue harvesting included the preparation of a one-cm-thick transverse slice through the cardiac ventricles midway between apex and atrioventricular groove. This slice was frozen at −80°C until further study, and the remainder of the heart was fixed in cold buffered 4 percent formaldehyde solution. On arrival at the laboratory, hearts were weighed and examined by a standard autopsy protocol (AHK). The thicknesses of LVW, RVW, and VS were recorded. National Disease Research Interchange (Philadelphia, PA, USA) provided 10 formalin-fixed and 8 frozen normal heart samples. In 14 cases, the FA mutation, a pathogenic homozygous guanine-adenine-adenine (GAA) trinucleotide repeat expansion, was known during life or determined by polymerase chain reaction on deoxyribonucleic acid (DNA) extracted from frozen cerebellar cortex. In case FA15 (Table 1), the GAA expansion was not determined during life. All tissues had been fixed in formaldehyde solution at the time of autopsy, precluding *post mortem* DNA analysis. Morphological study of heart and nervous tissue, however, confirmed the diagnosis of FA.

### Quantitative XRF of Fe and Zn in LVW, RVW, and VS

**Fig 1:**
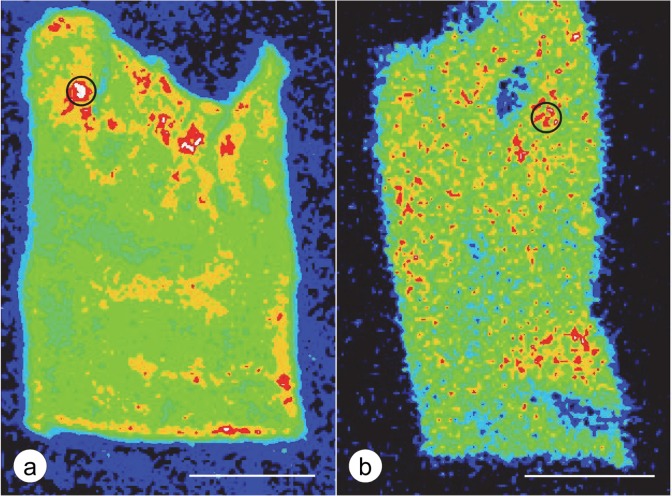
XRF mapping and quantitative *in situ* measurements of Fe. (a) LVW in FA (patient FA5, Table 1), (b) LVW in a normal control. Pseudocolors represent the intensity of Fe XRF. White indicates maximal emission. Red, orange, green, light blue, and dark blue represent progressively lower fluorescence, respectively. The distribution of Fe in FA and normal heart is heterogeneous. Circles with an area of 1 mm^2^ were placed over regions of strongest Fe XRF, and 25 point measurements were made inside the outlined area. Fe concentrations in μg/ml tissue volume were calculated by reference to PEG-encapsulated Fe-III- and Zn-II-mesoporphyrin as previously described [6,8]. In FA (a), the Fe-rich regions appear larger and show a more extended gradient into the surrounding tissue than the control (b). In FA (a), 25 repeated measurements in the circular area of 1 mm^2^ yields an average Fe concentration of 131.8 μg/ml tissue volume; in the illustrated normal control (b), the averaged Fe level is 33.7 μg/ml tissue volume. Quantitative Zn levels in the same outlined regions were obtained after switching to the Zn XRF "map". Levels are 24.3 μg/ml in FA (a) and 20.7 μg/ml in the control sample (b). Bars, 5 mm.

Progressive infiltration of tissue samples by PEG 400, PEG 1000, and PEG 1450, preparation of Fe-III- and Zn-II-mesoporphyrin standards; and *in situ* quantification of Fe and Zn were identical to the techniques described before [6, 8]. The XRF instrument was custom-assembled by X-Ray Optical Systems (East Greenbush, NY, USA) and consisted of a molybdenum X-ray tube operated at 48 KV and three doubly-curved crystal optics generating monochromatic radiation over a spot size of 0.06 mm^2^. The beam scanned the specimen in steps of 0.15 mm, and metal-specific fluorescence was collected as counts/13 sec. Twenty-five repeated measurements were made in Fe-rich regions of FA and normal LVW, RVW, and VS as described in the legend to Fig. 1. Results were expressed as μg metal/ml tissue volume, based on a validated protocol described previously [8]. After metal mapping and *in situ* quantification were complete, PEG was removed by immersion and repeated washing in phosphate-buffered saline (PBS). The PEG-free specimens were fixed in a phosphate-buffered solution of 4 percent paraformaldehyde (pH 7.4) for 4 h at 4°C and re-embedded in paraffin for subsequent slide techniques. This sequence allowed precise alignment of X-ray maps with stained tissue slides.

### Metal assay in bulk extracts of cardiac tissues

Frozen samples of LVW, RVW, and VS were weighed and transferred into polypropylene vials for analysis. After freeze-drying to constant weight, the dried tissues were again weighed prior to digestion at atmospheric pressure with double-distilled concentrated nitric acid in a microwave-assisted reaction system (CEM Corporation, Matthews, NC, USA). Acid digests were diluted 8-fold with deionized water and analyzed for Fe and Zn by inductively coupled plasma-optical emission spectrometry (ICP-OES), using a Perkin Elmer Optima 5300 DV instrument (Shelton, CT, USA). The analytes were measured at the respective wavelengths of 259.939 nm for Fe and 213.857 nm for Zn. Standard solutions were prepared from pure single metal stock solutions (purity of 99.999%; High Purity Standards, Charleston, SC, USA). The ICP-OES method was validated through the analysis of certified reference materials as described previously [9]. Results were calculated as μg metal/g dry weight and converted to μg/g wet weight based on the frozen water content.

### Frataxin assay by enzyme-linked immunosorbent assay (ELISA)

Frataxin levels were assayed in extracts of frozen LVW of 13 of the 15 FA cases shown in Table 1 and of 8 normal controls. Weighed samples of wet tissue of LVW (100–300 mg) were dispersed by repeated 5-sec-long bursts of ultrasonication in a lysis buffer containing 100 mM tris-HCl, pH 7.5, 150 mM NaCl, 1 percent each of the nonionic detergents Triton X-100 and Nonidet P-40 (vol/vol), 5 mM ethylenediamine tetraacetic acid, 5 mM ethylene glycol tetraacetic acid, and 1 percent protease inhibitor cocktail (vol/vol; Sigma, St. Louis, MO, USA) [10]. The mixtures were chilled on ice between bursts. Typically, 10 bursts were required to achieve complete homogenization of the sample. The homogenates were centrifuged for 2h at 14,000 x g at 4°C to obtain a clear extract. The supernatant was collected, and aliquots were diluted 1:10 in PBS to reduce the detergent concentrations to 0.1%. The diluted extracts were then filtered through centrifugal filter devices with a molecular weight cut-off of 30 kDa (EMD Millipore, Billerica, MA, USA) at 14,000 x g for 45 min. The filtrate was collected for ELISA of frataxin. Polystyrene ELISA plates (Santa Cruz Biotechnology, Santa Cruz, CA, USA) were coated with monoclonal anti-frataxin antibody (0.33 μg protein/ml; Abcam, Cambridge, MA, USA, catalogue number, Cat. No., ab110328) in 0.05 M carbonate buffer (pH 9.6) by an overnight incubation at 4°C. The plates were washed three times with a 1 percent solution of non-fat dry milk in PBS, containing 0.1 percent Tween 80 (NFDM-PBS-Tween 80). Well surfaces were then covered for 4h at room temperature with NFDM-PBS-Tween 80 to block non-specific absorption of antibodies. The next step was the application of diluted tissue lysate or recombinant human frataxin in NFDM-PBS-Tween 80. After an overnight incubation, the wells were drained and washed with NFDM-PBS-Tween 80. The detecting antibody was rabbit polyclonal anti-frataxin (whole serum; courtesy of Dr. Grazia Isaya) that was diluted 1:1000 in NFDM-PBS-Tween 80. After an overnight incubation at 4°C, the plates were washed 3 times with NFDM-PBS-Tween 80, followed by PBS. The wells were filled with biotinylated anti-rabbit IgG (0.75 μg protein/ml) in PBS and maintained at room temperature for 2h. After washing with PBS, the next step was a 1-h incubation at room temperature in a solution of horseradish peroxidase-labeled streptavidin (0.25 μg/ml). After washing with PBS, a chromogenic solution of ortho-phenylenediamine (2 mM) and hydrogen peroxide (0.01%) in 0.1 M citric acid-sodium phosphate buffer (pH 5.0) was added to each well. A distinct color gradient developed within 2–3 min, and the addition of 2.5 M sulfuric acid (50 μl) stopped the reaction. Absorbance at 492 nm was determined using an ELISA plate reader (SpectraMax Plus, Molecular Devices, Sunnyvale, CA, USA). The amount of frataxin in tissue lysates was determined by reference to a calibration standard curve, and results were expressed as ng/g original wet weight.

### Histochemistry, immunohistochemistry, and immunofluorescence

For Fe histochemistry, immunohistochemistry, and immunofluorescence, paraffin sections of 6 μm thickness were dewaxed by routine methods. Iron was visualized with Perls's reagents (a mixture of 1% hydrochloric acid and 1% potassium ferrocyanide, weight/vol), and sections were counterstained by Brazilin (Anatech, Battle Creek, MI, USA). For immunohistochemistry, the rehydration of tissue sections also included suppression of endogenous peroxidase by 30-min-long oxidation in 3 percent hydrogen peroxide in methanol (weight/vol). The following antibodies were available from commercial sources (suppliers and Cat. No. in parentheses): rabbit polyclonal anti-human liver ferritin (Immunology Consultants, Portland, OR, USA, Cat. No. RF-80G); rabbit polyclonal anti-α-actinin (Abcam, Cambridge, MA, USA, Cat. No. ab62298); mouse monoclonal anti-hepcidin (Santa Cruz, Santa Cruz, CA, USA, Cat. No. sc-100277); and mouse monoclonal anti-CD68 (Santa Cruz, Cat. No. sc-20060). A monoclonal antibody against mitochondrial ferritin was raised and purified at San Raffaele Scientific Institute, principally as described by Luzzago et al [11]. In tissue sections, the antibody, designated AL51, does not cross-react with the human heavy (H)-chain ferritin subunit though mitochondrial ferritin and H-chain ferritin share extensive amino acid sequence homology [12]. Absorption of the diluted antibody by a 10-fold excess of soluble heart ferritin (Lee Biosolutions, St. Louis, MO, Cat. No. 270–70) did not block immunoreactivity with tissue sections of FA myocardium. The specificity of anti-hepcidin was confirmed by preincubation of the antibody solution (0.4 μg/ml) with a 10-fold excess of human recombinant hepcidin (Abnova, Taipei, Taiwan, Cat. No. H00057817P01) for 4h at room temperature. This step effectively blocked immunohistochemical staining of cardiac monocytes and macrophages. Antigen retrieval steps varied with the antigen of interest (antigen in parentheses): 45 min-chelation in a solution of 2 mM each of 2,2'-dipyridyl and sodium hydrosulfite in acetic acid-sodium acetate buffer pH 6.0 (cytosolic and mitochondrial ferritins; hepcidin); incubation in diluted DIVA (1:10), a proprietary decloaking solution (Biocare Medical, Concord, CA, USA) for 30 min at 95°C (cytosolic and mitochondrial ferritins; hepcidin); incubation in 0.05 M citric acid-sodium citrate buffer (pH 4.6) for 10 min at 95°C, followed by cooling to room temperature for 10 min (CD68).

Concentrations of antibodies were optimized and ranged from 0.4 μg protein/ml (anti-hepcidin) to 16.62 μg protein/ml (anti-mitochondrial ferritin). The sequence of incubations for the visualization of immunohistochemical reaction products was as described before [6] and included amplification by biotinylated anti-rabbit (or mouse) IgG (0.6 μg protein/ml for DIVA-treated sections, 3 μg protein/ml for all others), horseradish peroxidase-labeled streptavidin (0.4 μg protein/ml for DIVA-treated sections, 2 μg protein/ml for all others), and diaminobenzidine/urea/hydrogen peroxide as the chromogen (Sigma).

For double-label immunofluorescence, the following "pairs" of antigens were processed: mitochondrial ferritin/cytosolic ferritin; hepcidin/cytosolic ferritin; and hepcidin/α-actinin. The initial steps included section rehydration and suppression of non-specific signals. Antigen retrieval and antibody dilution were the same as for immunohistochemistry. Oxidation in hydrogen peroxide-containing methanol was omitted. For monoclonal antibodies, the sequence of incubations was as follows (washing steps omitted): overnight incubation at 4°C in the first primary antibody; application of Alexa 488-labeled donkey anti-mouse IgG (2 μg protein/ml) for 4h at room temperature; re-suppression in 10% normal donkey serum; an overnight cold incubation in the second primary antibody, followed by incubation in Cy3-labeled donkey anti-rabbit IgG (2 μg protein/ml) for 4h at room temperature. After washing, the sections were covered by a solution of 50% glycerol in PBS. Alexa 488- and Cy3-labeled secondary antibodies were purchased from Jackson ImmunoResearch (West Grove, PA, USA). The sections were viewed in a laser scanning confocal microscope (Zeiss LSM 510 Meta). Exciting wavelengths were 488 and 543 nm for Alexa 488 and Cy3, respectively. The band pass filters were set at 500–530 nm for Alexa 488 and 565–615 nm for Cy3.

### Heart fiber size and numbers

**Fig 2:**
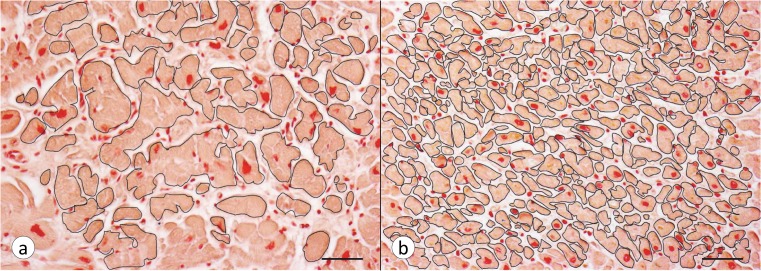
Fiber counts and cross-sectional areas in FA cardiomyopathy. (a) FA (patient FA2, Table 1), (b) normal control. Transverse Brazilin-stained sections of VS were photographed at a magnification of 200X, and analyzed for fiber density and cross-sectional area within a field of 0.15 mm^2^. (a) FA: The section shows paucity of fibers, hypertrophy, irregular contours, size variability, and endomysial thickening. The total number of fibers/0.15 mm^2^ is 73, corresponding to 487/mm^2^. The mean cross-sectional area is 804 μm^2^ (range, 37–3953). (b) Normal control: Fibers are much smaller. The total number of fibers/0.15 mm^2^ is 337, corresponding to 2247/mm^2^. The mean cross-sectional area is 249 μm^2^ (range, 24–664). Bars, 50 μm.

The Brazilin counterstain used for Fe histochemistry provided good contrast between heart fibers and surrounding endomysium (Fig. 2). Areas of LVW and VS representing cross-sections of stained cardiomyocytes were photographed at a magnification of 200 X. The cross-sectional area of each fiber was determined by a Zeiss AxioVision program (Carl Zeiss, Göttingen, Germany) as described in the legend to Fig. 2. Cardiomyocytes extending beyond the edge of the microphotograph were omitted. The computer program also generated a data table from which the number of fibers/field could be calculated. Sections of RVW did not contain an adequate number of transversely cut fibers and were not analyzed.

### Data analysis

Cross-sectional areas of cardiomyocytes were used to generate a Tukey box-and-whisker plot. For statistical analysis, area data were logarithmically transformed to correct skew and heteroscedasticity. The mean log area was calculated for each patient, and a two-tailed t-test was used to compare the averages between FA and controls. The geometric means and their 95% confidence intervals (95% CI) were computed by using the antilogarithm of transformed data. Differences between LVW, RVW, and VS of FA patients and control subjects obtained by measurement of Fe and Zn by *in situ* XRF (Table 2) and by ICP-OES of bulk digests (Table 3), respectively, were analyzed by two-tailed t test, assuming unequal variances.

**Table 2:** *In situ* quantification of Fe and Zn in the hearts of 15 patients with FA^a^ and 10 normal controls (XRF).

	LVW^a^	RVW^a^	VS^a^
Fe^a^			
**FA (15)**	108.6±56.6^b^	70.3±36.0	125±73.9
**Normal controls (10)**	57.3±28.2	52.7±26.3	61.8±32.2
**p-values** ^c^	0.007	0.173	0.008
**Zn** ^a^			
**FA (15)**	18.3±13.3	14.8±7.7	21.7±17.3
**Normal controls (10)**	21.2±7.5	17.1±6	20.7±6
**p-values** ^c^	0.486	0.416	0.825

^a^Abbreviations: FA, Friedreich ataxia; Fe, iron; LVW, left ventricular wall; RVW, right ventricular wall; VS, ventricular septum; Zn, zinc

^b^Results of Fe and Zn are expressed as mean μg metal/ml tissue volume ± standard deviation. Number of FA patients and normal controls are given in parentheses.

^c^p-values based on statistical comparison by two-tailed t-test at α = 0.05, assuming unequal variances

**Table 3:** Fe and Zn concentrations in the heart of 13 FA^a^ patients and 8 normal controls (bulk digests).

	LVW^a^	RVW^a^	VS^a^
Fe^a^			
**FA (13)**	73.4±22.0^b^	63.8±26.0	71.4±23.0
**Normal controls (8)**	57.4±16.2	47.9±10.6	54.1±14.8
**p-values** ^c^	0.057	0.066	0.05
**Zn** ^a^			
**FA (13)**	19.3±3.4	18.2±3.7	23.8±3.8
**Normal controls (8)**	21.9±4.6	17.5±3.7	24.6±2.8
**p-values** ^c^	0.196	0.698	0.599

^a^Abbreviations: FA, Friedreich ataxia; Fe, iron; LVW, left ventricular wall; RVW, right ventricular wall; VS, ventricular septum; Zn, zinc

^b^Results of Fe and Zn are expressed as mean μg metal/g wet tissue ± standard deviation. Number of FA patients and normal controls are given in parentheses. In one FA patient, only LVW tissue was available.

^c^p-values are based on statistical comparison by two-tailed t-test at α = 0.05, assuming unequal variances

## Results

### Clinical and pathological data of FA patients

The series in Table 1 does not include late-onset cases of FA (>24.4 years [13]), and the mean ages of onset and death are somewhat lower than those in the larger series of 41 from which the current cases were selected [1]. In 10 of the 15 FA patients listed in Table 1 the direct cause of death was heart failure. The most common pathology of FA heart disease was concentric hypertrophy with reduced ventricular sizes and thickened walls, but asymmetric cardiomyopathy with a dilated right ventricle was present in one case (FA3, Table 1). Death from endocarditis, sudden cardiac failure after scoliosis surgery, and brain embolism were also possible manifestations of FA heart disease. "Cachexia" as a cause of death in two patients (Table 1) was attributed to a fatal *neurological* course. Height and weight of the FA patients were not available, and body mass indices could not be calculated. The mean heart weights of the normal controls were higher than those reported from a large French forensic practice (women, 312±78 g, N = 329; men 365±71 g, N = 355) [14]. The mean respective thicknesses of LVW, RVW, and VS in mm ± S.D. were 18±3 (range: 13–23), 8±2 (range: 4–12), and 17±3 (range: 13–25). These measurements were all higher than the published means in a very large autopsy series [15]. Frataxin levels in the LVW were at or below the detection limit of ELISA (25 pg), representing a level of <15 ng/g wet weight. In LVW of 8 normal controls, mean frataxin concentration and standard deviation (S.D.) were 235.4±75.1 ng/g wet weight, ranging from 140 to 352. The very low levels of frataxin in the LVW of FA patients precluded correlation with the clinical and genetic parameters given in Table 1.

### Quantitative analysis of Fe in FA myocardium

All cases listed in Table 1 showed Fe-positive inclusions though the degree of fiber hypertrophy and fibrosis varied. Fig. 1 displays the heterogeneous distribution of Fe in the LVW of FA (Fig. 1A) and a normal control (Fig. 1B), and how zones were selected for repeated Fe and Zn measurements. Table 2 lists mean *in situ* Fe and Zn concentrations in LVW, RVW, and VS that were obtained from regions of highest Fe XRF on corresponding metal maps. The comparison of FA and normal controls shows significantly higher Fe levels in LVW and VS of FA, but not in RVW. In the same regions, Zn levels do not differ. Differences in Fe and Zn levels obtained by assay of bulk digests of normal and FA LVW, RVW, and VS were not significant though a trend toward higher Fe concentrations was evident (Table 3). The water content of FA heart tissues (in percent ± S.D.) was 79.0±4.4 for LVW, 77.3±6.1 for RVW, and 79.3±3.2 for VS. For normal controls, water content for LVW, RVW, and VS was 79.4±1.3, 77.3±5.2, and 79.5±1.3, respectively. Polyethylene glycol 1450 replaces all tissue water, and metal concentrations given as μg/ml tissue volume (XRF) can be converted to μg/g wet tissue by assuming a density of 1 and multiplying values by the water content.

### Cardiomyocyte hypertrophy in FA

**Fig 3:**
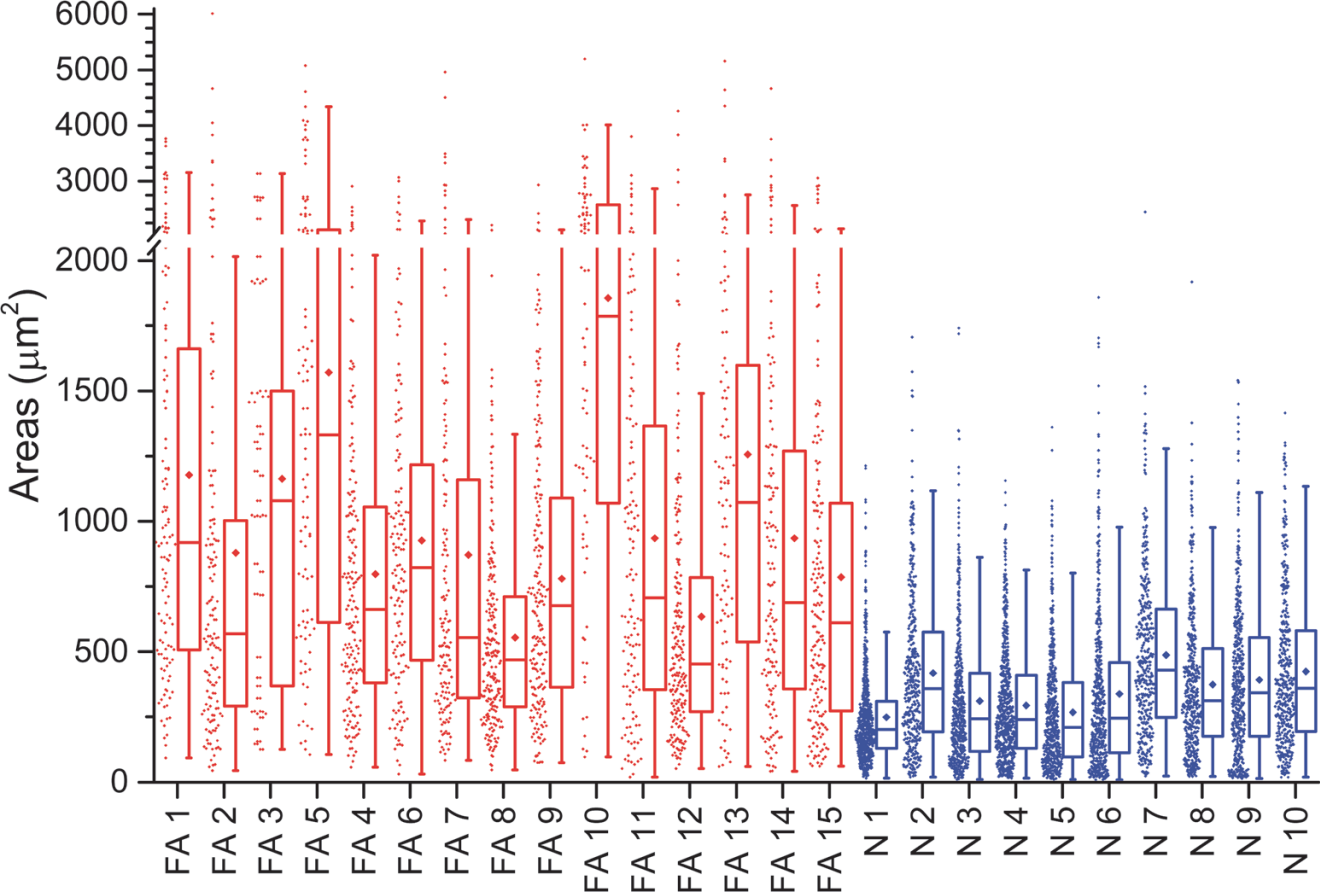
Fiber hypertrophy in FA cardiomyopathy. A Tukey box-and-whisker plot together with the raw data points to the left of each box, shows fiber size distribution in the LVW of 15 patients with FA (FA1-FA15, corresponding to Table 1) and 10 normal controls (N1-N10). FA cases are shown in red, normal controls in blue. In the box plot, the central rectangle spans the first quartile to the third quartile (the interquartile range), and the horizontal line within the rectangle marks the median. The diamond symbol indicates the mean. The "whiskers" above and below the box are drawn to the furthest point within 1.5 x IQR from the box (the non-outlier range). The range of fiber sizes in FA is much larger than in normal controls. All medians and means in FA are higher than in normal controls. See text for statistical analysis of the data.

Fig. 2 displays transverse sections of VS in FA (Fig. 2A) and a normal control case (Fig. 2B). Hypertrophy in FA hearts is defined by abnormally large cross-sectional areas of cardiomyocytes. Beyond hypertrophy, however, the images also show unusual contours and an overabundance of endomysial connective tissue. Fig. 3 presents a Tukey box-and-whisker plot of fiber sizes in the LVW of 15 cases of FA and 10 normal controls. The plot for VS is very similar (not displayed). All fiber size medians and means in fields of 0.3 mm^2^ are larger in FA than in normal controls. The geometric mean of the cardiomyocytes of the LVW area in 15 FA patients is 693 μm^2^ (95% CI: 579–829) and significantly greater than the mean area in 10 normal controls, which is 250 μm^2^ (95% CI: 211–296) (p<0.001). The geometric mean of cardiomyocytes in the VS of 15 FA patients, 526 μm^2^ (95% CI: 446–620), is also significantly greater than the mean in 10 controls (233 μm^2^, 95% CI: 191–285) (p<0.001).

### Fe, cytosolic ferritin, and mitochondrial ferritin in FA myocardium

**Fig 4:**
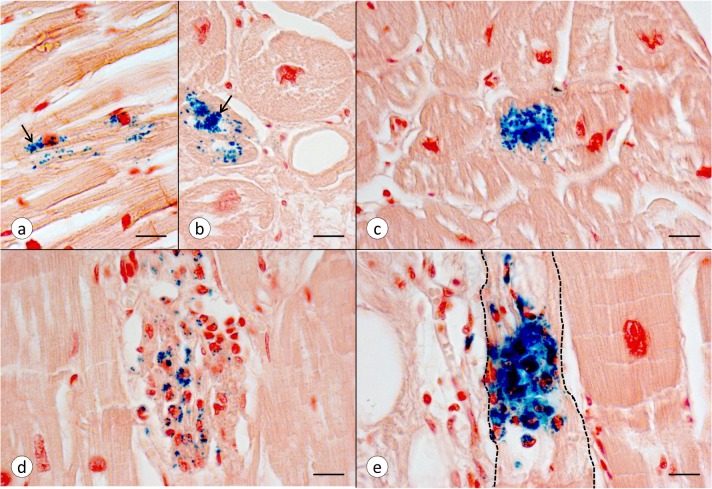
Various stages of Fe accumulation in FA cardiomyopathy. All microphotographs derive from LVW of patient FA7 (Table 1). (a) Punctate Fe reaction product lies parallel to the long axis of a cardiomyocyte and near two nuclei (arrow). The fiber is otherwise intact. (b) The transverse section of a heart fiber shows similar punctate Fe reactivity but also a region of Fe aggregation (arrow). (c) This fiber shows an advanced stage of Fe accumulation and aggregation. (d) This image represents an inflammatory nodule with macrophages containing granular Fe. (e) Fe-laden phagocytes invade and replace sarcoplasm and myofibrils of a single fiber. The involved cardiomyocyte is outlined by interrupted lines. Fe stain, Brazilin counterstain. Bars, 20 μm.

Fig. 4 illustrates various stages of Fe accumulation in the LVW of FA cardiomyopathy. The most common pattern consists of small Fe-positive granules that lie parallel to the long axis of the fiber (Fig. 4A). Fig. 4B and C shows greater aggregation of Fe reaction product though the affected fibers appear otherwise intact. In Fig. 4D, Fe is localized to the cells of a nodular infiltrate, but it is not at once apparent that the nodule has replaced a cardiomyocyte. Fe accumulation in phagocytes and fiber destruction, however, are evident in Fig. 4E. Normal heart sections never show Fe-rich inclusions.

**Fig 5:**
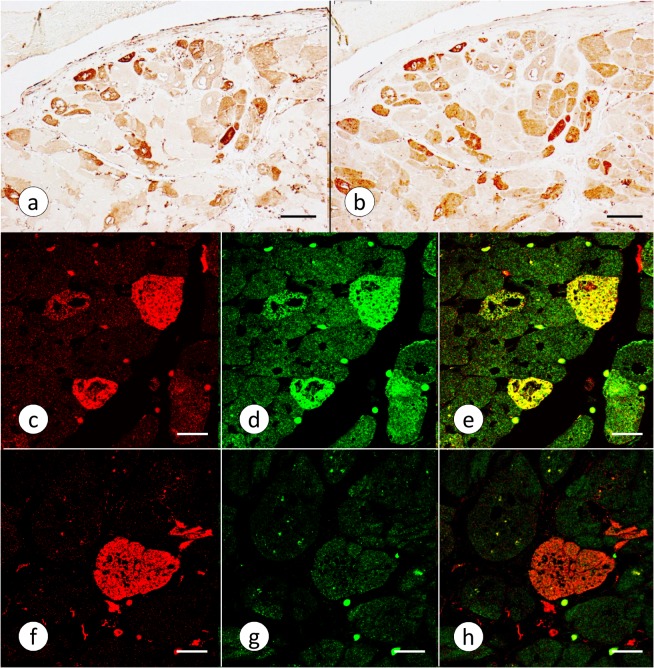
Cytosolic and mitochondrial ferritin in FA cardiomyopathy. (a) and (b), positive contrast immunohistochemistry of adjacent sections (LVW, FA patient FA10, Table 1); (c-h), laser scanning confocal immunofluorescence (LVW, FA patient FA7, Table 1). (a), cytosolic ferritin; (b) mitochondrial ferritin; (c) and (f), cytosolic ferritin visualized by Cy3 (red); (d) and (g), mitochondrial ferritin visualized by Alexa 488 (green); (e) and (h) are merged images of (c) and (d), and (f) and (g), respectively. Cytosolic (a) and mitochondrial ferritin (b) show extensive co-localization of their immunohistochemical reaction products. In the series (c-e), three heart fibers display co-localization of cytosolic and mitochondrial ferritin. In the series (f-h), a single fiber is strongly fluorescent for cytosolic ferritin but does not express mitochondrial ferritin. Bars: (a-b), 100 μm; (c-h), 20 μm.

The available antibody to human liver ferritin reveals distinct reaction product in many cardiomyocytes of FA (Fig. 5A). It yields no reaction product in normal human cardiomyocytes (not illustrated). The monoclonal antibody to mitochondrial ferritin shows extensive co-localization with cytosolic ferritin in cardiomyocytes (Fig. 5B). Mitochondrial ferritin is only rarely expressed in inflammatory cells. When present, it may reflect phagocytosis of cardiomyocytes that biosynthesize an excess of mitochondrial ferritin. Sections of normal heart do not yield reaction product with anti-mitochondrial ferritin. The confocal images in Fig. 5C-E confirm co-localization of mitochondrial and cytosolic ferritin. One fiber that fluoresces brightly for cytosolic ferritin (Fig. 5F), however, appears devoid of mitochondrial ferritin (Fig. 5G).

### Inflammation in FA cardiomyopathy

**Fig 6:**
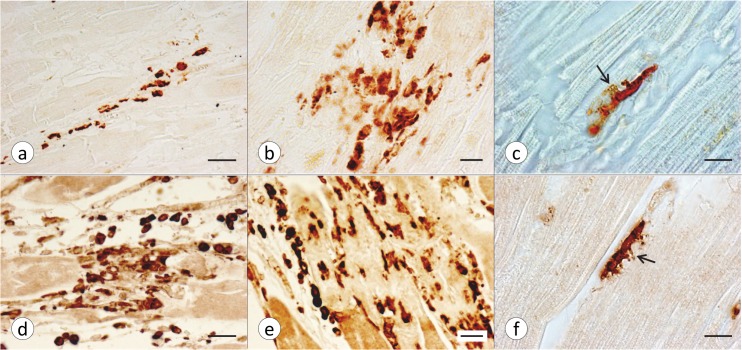
The inflammatory infiltrate in FA cardiomyopathy. Immunohistochemistry of CD68 (a-c) and hepcidin (d-f). All sections derive from LVW of FA patients in Table 1: FA5 (a-b), FA8 (c), FA7 (d-e), FA13 (f). The microphotographs in (c) and (f) were taken under differential interference optics to improve contrast, visualize cross-striations of cardiac muscle, and highlight fiber invasion by pseudopods of monocytes (arrows). The cellular infiltrate may be restricted to the endomysium (a) but is most intense following fiber invasion (b and e). Fiber invasion seems to begin with close attachment and breaching of the plasma membrane by delicate CD68- or hepcidin-positive processes, respectively (c and f, arrows). Bars: (a) and (d), 50 μm; (b) and (e), 20 μm; (c) and (f), 10 μm (oil immersion).

**Fig 7:**
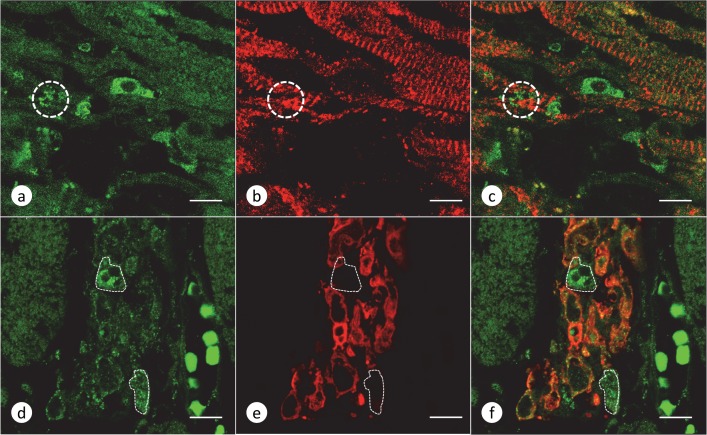
Hepcidin and cytosolic ferritin in FA myocarditis. LVW of patient FA7 (Table 1). Double-label laser scanning confocal immunofluorescence of hepcidin (Alexa 488 green, a and d), α-actinin (Cy3 red, b), and cytosolic ferritin (Cy3 red, e); (c) and (f) are merged images of (a-b) and (d-e), respectively. (a-c) Invasion of a cardiomyocyte by hepcidin-reactive cells. The circles in (a-c) indicate hepcidin-reactive processes among remaining α-actinin-reactive Z-discs. (d-f) Inflammatory cells in the endomysium. Most inflammatory cells contain both hepcidin and cytosolic ferritin (d-f). The interrupted lines indicate hepcidin-reactive cells that display no ferritin fluorescence. Bars: 10 μm.

Based on "Dallas criteria" [16], FA cardiomyopathy is consistent with myocarditis. In all 15 cases of FA (Table 1), the myocardium contained an abnormal number of CD68-reactive monocytes (Fig. 6A-C). Immunohistochemistry with an alternate marker of monocytes and macrophages, CD14, gave a similar result (not illustrated). Frank fiber invasion (Fig. 6B) was observed in 10 cases, and some images showed attachment of monocytes and penetration of the plasma membrane by short pseudopods (Fig. 6C). Polymorphonuclear leukocytes, CD3-, or CD20-reactive lymphocytes were absent. Many cells in the inflammatory infiltrate also displayed immunoreactivity with anti-hepcidin (Fig. 6D-F), and Fig. 6F illustrates attachment of a hepcidin-positive cell extending pseudopods into the interior of a fiber. Hepcidin-containing cells participate in the invasion of cardiomyocytes (Fig. 7A-C), and most but not all cells in the inflammatory infiltrate of the endomysium are reactive for ferritin *and* hepcidin (Fig. 7D-F).

## Discussion

### Limitations of the study

This study of autopsy samples determined abnormalities at advanced or late stages of FA. Disease durations ranged from 4 to 54 years (Table 1; mean ± S.D., 27±12 years). Morphological and biochemical findings cannot necessarily be extrapolated to the status of FA cardiomyopathy at earlier stages in a given FA patient. Case FA11 (Table 1) with a disease course of only 4 years, however, suggests that myocarditis in FA is not the result of protracted illness. The very low cardiac frataxin levels at the time of death also do not permit conclusions about the possibility that concentrations of this protein might have been higher at a time when the disease was less advanced. Diagnostic endomyocardial biopsies in young FA patients are no longer justified, but it is known that cardiac Fe accumulates very early in the disease (FA11, Table 1 and [5]). A patient reported in ref. [5] had a heart biopsy at the age of 9 years to establish the reason for her cardiomyopathy. The diagnosis of FA was made only later, and she succumbed to the disease at the age of 26 years. A comparison of biopsy and autopsy samples revealed very similar Fe excess. The biopsy showed no endomysial fibrosis whereas the autopsy tissues displayed extensive scarring.

### Fiber hypertrophy in FA

Fiber hypertrophy (Fig. 2) may be an adaptive response to fiber loss, but large lobulated fibers in FA (Fig. 2A) may also be due to coalescence of smaller adjacent fibers. Sizing and counting of fibers do not fully capture the extensive remodeling of the heart in FA because these methods do not record cardiomyocyte length, the degree of branching, changes in myofibrils, and the extent of endomysial scarring. In a separate communication, we will present the major modification of intercalated discs and gap junctions in FA cardiomyopathy. The "signaling pathways" for cardiac hypertrophy and the many involved biomolecules have attracted attention for several years [17–20], but the cited studies did not consider frataxin deficiency and how it might cause heart disease in FA. At the time of two of the earlier reports [17–18], the importance of frataxin in FA was still emerging.

### Fe and ferritin species in FA cardiomyopathy

FA cardiomyopathy is unlike hemochromatosis in which the heart can accumulate very large amounts of the metal [21] before manifesting cardiac failure. It is not clear why the restricted accumulation of cardiac Fe in FA should be much more damaging than global Fe overload. The various types of Fe accumulation occur in close proximity and are thought to reflect progression from small granules to coarse aggregates (Fig. 4A-C). Aggregation is most likely due to over-abundant ferritin, resembling the formation of siderin or hemosiderin. The results described here do not establish the precise mechanism by which Fe gains access to the interior of cardiomyocytes. The involved heart tissue responds to Fe entry by biosynthesizing cytosolic ferritin (Fig. 5A, C, and F) and mitochondrial ferritin in the same cell (Fig. 5B and D) though the biochemical mechanisms are very different. Fe stimulates the biosynthesis of cytosolic ferritin by interacting with an iron-responsive element in the cognate messenger ribonucleic acid (mRNA). In contrast, mRNA of mitochondrial ferritin does not contain an Fe-responsive element [22], and accelerated biosynthesis in the presence of Fe occurs at the level of transcription. From experiments with transfected HeLa cells, Drysdale et al [22] concluded that Fe incorporation into cytosolic and mitochondrial ferritin was equally efficient. The observations also imply that Fe entry into mitochondria is not a rate-limiting step. In analogy, the co-localization of cytosolic and mitochondrial ferritin in FA cardiomyopathy (Fig. 5) is more consistent with a pancellular Fe excess rather than selective mitochondrial Fe accumulation caused by frataxin deficiency [23]. The relative extent of cytosolic and mitochondrial Fe excess in FA hearts is unknown. Heart transplantation is emerging as a therapeutic option in FA cardiomyopathy, and it is conceivable that mitochondria may be isolated from the explant for Fe assay and other biochemical analyses. The exemption of the RVW from significant regional Fe excess in FA (Table 2) differs from previously published data that showed higher focal Fe levels in five anatomical sites of the heart including RVW [6]. The reason is likely technical: The method described here quantifies Fe by *in situ* XRF over a much larger region (1 mm^2^) (Fig. 1). A comparison of XRF (Table 2) and ICP-OES of Fe (Table 3) confirms that FA does not cause diffuse cardiac Fe excess, as previously reported after colorimetric assay of the metal [5]. The assay of Zn was included in XRF and ICP-OES as a baseline to highlight the selective increase of Fe. XRF does not distinguish Fe in small granular inclusions in cardiomyocytes (Fig. 4A-C), inflammatory infiltrates (Fig. 4D), or phagocytized fibers (Fig. 4E). Retention of Fe in inflammatory cells may be more important in the pathogenesis of FA cardiomyopathy than the small granular inclusions in cardiomyocytes or the amount of total heart Fe.

### Fe excess and myocarditis

Regional Fe accumulation cannot be the sole mechanism in the pathogenesis of FA myocarditis. Cardiac Fe excess in hemochromatosis causes fibrosis without inflammation [21]. Attachment of monocytes to, and penetration of, cardiomyocyte plasma membranes (Fig. 6) and strong Fe expression in the inflammatory infiltrate (Fig. 4) may be unique for FA cardiomyopathy. Expression of hepcidin in FA myocarditis and attachment of a hepcidin-containing monocyte to a heart fiber (Fig. 6F) suggest that the Fe-regulatory protein causes Fe excess due to interaction with ferroportin, the principal Fe exporter [24]. Therefore, failure of Fe export had to be considered in the accumulation of Fe. In support of this mechanism, Ramirez et al [6] detected a paucity of ferroportin in FA cardiomyocytes that were fully involved in the accumulation of Fe. A systematic search, however, did not confirm the presence of monocytes abutting the plasma membrane of all cardiomyocytes with Fe-positive granules, and failing export of Fe from heart fibers due to local hepcidin biosynthesis may not adequately explain the accumulation of the metal. Hepcidin, a peptide hormone largely synthesized by the liver, controls systemic Fe distribution by gaining access to organs through blood flow. The protein is also present in non-hepatic tissues, including heart [25] and inflammatory cells [26]. Hepcidin responds primarily to the Fe needs of the entire body, but biosynthesis of this protein is also strongly stimulated by natural or experimental inflammation, principally mediated by interleukin 6 (IL-6) [27–28]. The significance of this cytokine for myocarditis in FA has yet to be determined.

Cytosolic ferritin is a marker of Fe excess, and its co-localization with hepcidin (Fig. 7D-F) may be the most evident signal of Fe dysmetabolism in FA hearts. The presence of hepcidin in the inflammatory infiltrate implies that the heart cannot discharge the metal from macrophages. It is peculiar that Fe toxicity in FA cardiomyopathy is similar to the instability of human and experimental atheromatous plaques [29–30]. The cited authors attributed the damaging effect of heme-derived Fe in atheroma to local hepcidin production and internalization of ferroportin. Fe-overloaded macrophages were thought to be the source of toxic Fe that affects surrounding tissues.

### Therapeutic considerations

A promising approach toward the replenishment of frataxin in FA patients with cardiomyopathy is the intravenous delivery of an adeno-associated virus vector expressing frataxin [31]. Such treatment of a conditional mouse model with FA cardiomyopathy is able to block onset of heart failure and reverse it after it has already been established. Assuming that hepcidin expression in myocarditis is a response to IL-6, it might be possible to improve FA cardiomyopathy by blockade of IL-6 signaling [32]. An advantage of anti-IL6 or anti-IL6-receptor therapy is the knowledge gained from previous clinical trials for the treatment of rheumatologic and neoplastic disorders.

## Conclusions

FA causes cardiomyocyte hypertrophy and extensive endomysial fibrosis. In contrast to cardiac hemochromatosis, the accumulation of Fe in cardiomyocytes is restricted to small regions of 1 mm^2^ or less, and short gradients of declining Fe involve the immediately adjacent myocardium. The process of Fe accumulation ranges from minute granules in heart fibers to coarse aggregates in cardiomyocytes undergoing phagocytosis. A main feature of FA cardiomyopathy is chronic myocarditis. The cells of the endomysial infiltrate are strongly reactive with antibodies to CD68 and hepcidin, supporting their monocyte/macrophage immunophenotype. Inflammatory cells in the endomysium, attachment of monocytes to the plasma membrane of cardiomyocytes, and necrosis of heart fibers are consistent with the "Dallas criteria" of myocarditis. The cardiac lesion of FA occurs on a background of extremely low frataxin levels, and restoration of this small mitochondrial protein and targeted anti-inflammatory therapy may benefit patients with FA.
